# Essential Oils-Loaded Polymer Particles: Preparation, Characterization and Antimicrobial Property

**DOI:** 10.3390/polym11061017

**Published:** 2019-06-09

**Authors:** Francesca Froiio, Lorianne Ginot, Donatella Paolino, Noureddine Lebaz, Abderrazzak Bentaher, Hatem Fessi, Abdelhamid Elaissari

**Affiliations:** 1 Department of Experimental and Clinical Medicine, University “Magna Græcia”of Catanzaro, Campus Universitario “S. Venuta”, Building of BioSciences, I-88100 Catanzaro, Italy; f.froiio@unicz.it (F.F.); paolino@unicz.it (D.P.); 2 Inflammation and Immunity of the Respiratory Epithelium-EA 7426, Faculté de Médecine Lyon Sud, 69495 Pierre Benite, France; azzak.bentaher@inserm.fr; 3 Univ Lyon, University Claude Bernard Lyon-1, CNRS, LAGEP-UMR 5007, F-69622 Lyon, France; lorianne.ginot@free.fr (L.G.); noureddine.lebaz@univ-lyon1.fr (N.L.); hatem.fessi@univ-lyon1.fr (H.F.)

**Keywords:** sweet orange essential oil, bergamot essential oil, antimicrobials, *Escherichia coli*, Eudragit RS100, nanoparticles, nanoprecipitation

## Abstract

In the last few years, essential oils (EOs) derived from plants have aroused great interest due to their well-known antimicrobial activity. Unfortunately, they present several limitations in their use, such as photosensitivity, temperature sensitivity, high volatility, and poor water solubility. The encapsulation technique represents a good solution to these problems and ensures protection of the functional properties of essential oils. In this work, bergamot essential oil (BEO) and sweet orange essential oil (OEO) loaded-Eudragit^®^ RS 100 (EuRS100) nanoparticles (NPs) were prepared by using the nanoprecipitation technique. We obtained nanoparticles characterized by a mean diameter of 57 to 208 nm and a positive surface charge (39 to 74 mV). The antibacterial activity of the obtained systems against *Escherichia coli* was in vitro investigated. We demonstrated that both orange and bergamot essential oils were successfully encapsulated and our nanoparticles have good antibacterial activity. Finally, in order to evaluate the potential applicability of OEONps to prolong fresh orange juice shelf-life, survival of *E. coli* during a storage period of one week at 25 °C was investigated: Orange essential oil-loaded nanoparticles (OEONPs) have been able to prolong the orange juice shelf life.

## 1. Introduction

The antimicrobial activity of natural essential oils and their compounds against food pathogenic microorganisms has been known for centuries [1,2]. *Citrus bergamia* (bergamot) and *Citrus sinensis* (sweet orange) are two plants belonging to the Rutaceae family. More than 90% of bergamot is produced in the southern coast of the Calabria region (south of Italy). BEO is applied in food, cosmetic, perfumery, and confectionery fields [3].

In literature, there are a lot of scientific works in which the antibacterial efficacy of *Citrus* essential oils has been proven [4,5,6,7]. Deans and Ritchie, and other authors have shown the antimicrobial activity of *Citrus* essential oils against several food-poisoning bacteria [8,9,10,11]. Tao et al. demonstrated orange essential oil antibacterial activity against some of the main bacteria responsible for food spoilage, like *Escherichia coli*, *Bacillus subtilis*, *Staphylococcus aureus* and *Saccharomyces cerevisiae* [7]. 

In another recent work, Guo et al. showed a strong antimicrobial activity of *Citrus sinensis* essential oil against *Escherichia coli*, *Staphylococcus aureus*, *Bacillus subtilis*, *Saccharomyces cerevisiae* [10]. 

Encapsulation of EOs is a good technique for enhancing their antimicrobial activity and for protecting essential oils and their compounds from degradation [12]. Eudragit RS100 (EuRS100), a methacrylate polymer, has been extensively used for developing nanoparticles [13].

Nanoprecipitation, also known as antisolvent precipitation, is a simple and reproducible technique patented by Fessi et al. in 1989 [14]. The nanoprecipitation technique, used for the encapsulation of both hydrophobic and hydrophilic compounds, offers many advantages compared to other techniques used for obtaining nanoparticles: (a) The nanoparticles are formed spontaneously, (b) the process is easily scalable, (c) it avoids the use of high quantities of toxic solvents and (d) high energy input is not necessary [15,16,17]. *Escherichia coli* is one of the main microorganisms responsible for food contamination and can cause serious diseases in humans [18,19]. Orange juice is one of the most consumed beverages in the world. Some microorganisms can contaminate natural orange juice, despite being kept under refrigeration. For this reason, natural orange juice has a short shelf life. To prolong the orange juice shelf life, different techniques are employed, such as pasteurization and conventional thermal treatment, but all of them cause a decrease of orange juice quality in terms of nutritional value (loss of vitamin C, for example), physicochemical properties and sensory characteristics [20,21]. The aim of this work was the encapsulation of bergamot and sweet orange essential oils in Eudragit RS-100 nanoparticles using the nanoprecipitation technique. The encapsulation efficiency, the physical-chemical properties of the obtained systems were investigated and their antimicrobial activity on *E. coli* was studied.

## 2. Materials and Methods 

### 2.1. Materials

Eudragit RS-100, a copolymer of ethylmethacrylate, methylmethacrylate and methacrylic acid esterified with quaternary ammonium groups (average molecular weight = 407,932 g/mol, ≥98% purity) was obtained from Evonik (Evonik Röhm GmbH, Darmstadt, Germany), acetone (≥99%), ethanol (≥99.8%) were obtained from VWR Chemicals (Prolabo, Paris, France). Bergamot essential oil was kindly provided by Bergamotto (Reggio Calabria, Italy) and sweet orange essential oil was kindly provided by Satyroi Coop.Soc. a r.l. (Reggio Calabria, Italy). They were obtained by cold pressing the peel of the fruits. Dulbecco’s Phosphate-Buffered Saline (DPBS+) was purchased from Thermofisher Scientific. Deionized water from Milli-Q system was used in all experiments.

### 2.2. Methods

#### 2.2.1. Gas Chromatography of *Citrus* Essential Oils

Analysis of BEO and OEO was carried out by Shimadzu system composed of GC-2010 equipped with capillary columns flame ionization detector (GC-FID). The gas chromatograph column is packed with the polyimide-coated fused silica phase. A GC Solution Software (Shimadzu, Milan, Italy) was used for the acquisition of the data. An Agilent 7890 GC apparatus equipped with a split/splitless injection and an FID detector, maintained at 250 °C, was used. As previously reported by Badri et al. (2018), a volume of 1 µL BEO/OEO was injected in split mode (1/120) to the HP-1 GC column (50 m × 0.320 mm × 0.50 µm). During this procedure, helium, used as a carrier gas, was maintained at constant pressure (3 psi). During the analysis, the oven temperature was modulated as follows: 8 min at 80 °C, increasing to 220 °C at 2 °C/min, from 220 to 310 °C at 10 °C/min and finally held isothermally 10 min at 310 °C [22]. 

#### 2.2.2. Identification of *Citrus* Essential Oils Components by GC-FID

GC-FID peak areas obtained from the capillary column were used to determine percentages of chemical compounds present in BEO and OEO by comparing their GC retention indices (RI) with that of literature data [23,24,25,26].

### 2.3. Nanoparticles Preparation *via* Nanoprecipitation Process

The nanoparticles were prepared by the nanoprecipitation technique described by Fessi et al. [14]. Two miscible phases were prepared: An aqueous phase and an organic one. The organic phase was obtained by dissolving 200 mg of Eudragit RS 100 in 25 mL of acetone. The aqueous phase consisted of 50 mL of distilled water.

The organic phase was added to the aqueous phase, drop by drop using a syringe, at room temperature and under moderate magnetic stirring (500 rpm). The evaporation of the organic solvent was performed subsequently using Buchi Rotavapor R-124^®^ (under low temperature and reduced pressure conditions). To obtain EOs-loaded nanoparticles, different amounts (5, 10, 25, 50, 100, 200, 300, 400 mg) of each essential oil were solubilized in the organic phase before being added to the aqueous one.

### 2.4. Orange Juice Preparation

In order to prepare orange juice samples, oranges were purchased from a local market, and they were used without undergoing any treatment. The juice was obtained by squeezing oranges in aseptic condition and then filtered by using fiberglass to remove solid particulate.

### 2.5. Nanoparticles Characterization

All prepared nanoparticle dispersions were characterized in terms of hydrodynamic size, zeta potential, morphology, colloidal stability (after storage at a different temperature), fluorescence microscopy and for EOs-loaded nanoparticles, encapsulation efficiency. 

#### 2.5.1. Hydrodynamic Size and Zeta Potential

Hydrodynamic size and zeta potential of prepared nanoparticles were measured using Zetasizer (ZetaNanosizer, Malvern Instruments limited, France), at a scattering angle of 90° and using a 4.5 mW laser diode operating at 670 nm as a wavelength source. The hydrodynamic size was calculated from the Strokes–Einstein diffusion coefficient measurement [27]. Helmholtz–Smoluchowski equation was used for the calculation of zeta potential values [28]. Colloidal stability studies of colloidal dispersions as a function of time was performed as follow: Each sample was divided into three aliquots of about 15 mL and stored at 4, 25, and 40 °C respectively. Size and zeta potential were then measured after one week, two weeks, and three weeks of storage. Results are the average of three measurements ± standard deviation. Before each measurement, samples were diluted in filtered 1 mM NaCl solution (1:100).

#### 2.5.2. Colloidal Stability Study

Colloidal stability study was investigated as a function of pH. Each dispersion (Empty NPs, BEO NPs, and OEO NPs) was diluted in NaCl at a given pH (from 2 to 12) and salinity (from 10^−4^ M to 10^−1^ M NaCl) before performing measurements.

#### 2.5.3. Fluorescence Microscopy Analysis

Fluorescence microscopy study was performed to verify BEO and OEO encapsulation in polymeric nanoparticles. Fluorescence microscope (Zeiss Axioplan 2 Imaging apparatus) equipped with ×10 and ×40 lenses and a monochrome camera, was used to obtain fluorescent images. A fluorescent molecule, quantum dots (QDs-PPs), was added to EOs before their encapsulation in EuRS100 based particles: This allowed to visualize if the essential oil was homogeneously encapsulated in the nanoparticles. The samples were prepared as follow: 5 µL from each sample (nanoparticles with and without EOs) was deposited onto a glass slide, dried at room temperature and observed by fluorescence light.

Each sample was excited firstly with a 550 (±25) nm band-pass filter (green light) and fluorescence from the sample (red color) was observed with a 605 (±70) nm band-pass filter. The analysis was done by comparing the fluorescence of the images obtained with the same focus.

#### 2.5.4. Scanning Electron Microscopy (SEM)

The nanoparticles morphology was investigated using Scanning electron microscopy (SEM). This analysis was performed with an FEI Quanta 250 FEG microscope at the “Centre Technologique des Microstructures” (CTμ) at the University of Lyon (Villeurbanne, France). Nanoparticles aqueous suspension was diluted (1:100). A drop of this solution was deposited on small steel support and left to dry at room temperature before analysis. Each sample, after being fixed on a standard sample holder, was sputter-coated with gold. Accelerating voltage of 15 kV was used to observe the samples. 

#### 2.5.5. Atomic Force Microscopy (AFM)

Atomic Force Microscopy (AFM) was performed using a Nano-observer, CSI Company (France). AFM Nano-Observer has XY scan range 110 µM (tolerance +/− 10%), Z range 9µm (tolerance +/− 10%) and XY drive resolution 24-bit control −0.06 Angstroms. A quantity of 10 µL of sample 1 was firstly drop cast onto silicon nitride substrate and then particles were scanned with AFM using silicon cantilever tip (ScienTec AppNano) of size L: 125 µm, W: 35 µm and T: 4.5 µm. The tip radius < 10 nm, H: 14–16 µm and with a frequency of 200–400 kHz and spring constant of K: 25–75 N/m. The scanning images were performed at 4 V amplitude, 3.8 V set point and Tip DC at 0 V. Measurements were performed in taping mode with a speed of 1 line per second and 512 resolution. Samples were analyzed in a (2 µm × 2 µm) area.

#### 2.5.6. Encapsulation Efficiency

##### Encapsulation Efficiency Determination Using Gas Chromatography

In order to determine the encapsulation efficiency percentage (EE%) and drug loading percentage, nanoparticles-loaded essential oils were centrifuged at 14,000 rotations per minute (rpm) for 30 min. Centrifugation allows the separation of the supernatant containing non-encapsulated essential oils from the nanoparticles-loaded essential oils. Then, limonene and myrcene for OEONps and limonene, linalool, linalyl acetate and terpinene for BEO Nps were quantified using gas chromatography (GC) equipped with flame ionization detector (GC-FID). For the analysis, Shimadzu GC 2010 plus equipped with split/splitless injector and capillary column Equity-5™ (30 m × 0.25 mm, 0.25 μm) conditioned at 250 °C for 30 min, was used. Helium (He) at a constant flow rate of 1 mL/min with a pressure of 65.3 kPa was used as a carrier gas, H2 flow was 30/30 mL/min and the airflow was 300/300 mL/min. During the analysis, the FID detector temperature was maintained at 300 °C and 2 μL of the sample was injected [22]. The oven temperature was increased from 100 to 180 °C at 2.5 °C/min. To determine the EE%, the following equation was employed:

(1) $$\mathrm{ncapsulation}\;\mathrm{efficiency}=\frac{\mathrm{Amount}\;\mathrm{of}\;\mathrm{encpauslated}\;\mathrm{drug}\;}{\mathrm{Initial}\;\mathrm{amount}\;\mathrm{of}\;\mathrm{drug}\;\mathrm{used}\;}\times 100$$

The pellet collected after centrifugation was dissolved in ethanol and then EE% and drug loading were determined. The drug loading % was defined as the amount of encapsulated drug in relation to the quantity of polymer used. Before the EE% and drug loading % determination methods were validated.

##### Encapsulation Efficiency Determination Using UV Spectrophotometer 

For UV analysis, a method reported by Prata and Grosso (2015) was followed [29]. Briefly, an exact concentration of essential oil in absolute ethanol was scanned in the range of 200–400 nm wavelength using UV-VISIBLE spectrophotometer: Maximum absorbance (λ_max_) was recorded at 327.5 nm wavelength for OEO and 311.5 nm for BEO. The calibration curves (absorbance versus essential oil concentration) were then established for each essential oil, and the following linear equations (R^2^ = 0.9989 for OEO (Equation (2)) and R^2^ = 0.9993 for BEO (Equation (3)) were obtained:

(2) Abs=0.0854×conc+0.0083

(3) Abs=0.9878×conc+0.1862

where *Abs* is the absorbance and *conc* is the essential oil concentration. 

Essential oil EE% was determined in the following way: 1 mL of a given sample was introduced in Amicon-Ultra filter-0.5 mL and then centrifuged at 14,000 rpm during 30 min. Supernatant was separated from pellet. The amount of encapsulated essential oil was determined after pellet dissolution in ethanol. Essential oil encapsulation efficiency (EE%) was directly determined by using the established calibration curve (Equations (2) and (3)) and the following equation:

(4) $$\mathrm{EE}=\frac{M}{\mathrm{Mo}}\times 100$$

where M (mg) is the amount of essential oil loaded in nanoparticles and Mo (mg) is the initial amount of essential oil added to the organic phase. All the measurements were carried out in triplicate. The UV-obtained data were compared with those obtained from gas chromatography (GC) analysis.

### 2.6. Antibacterial Activity Study

The antibacterial activity of essential oil-loaded nanoparticles was investigated against *Escherichia coli* bacteria. Overnight *E. coli* cultures were diluted (1:2) and grown aerobically in Luria–Bertani broth (10 mL) at 37 °C, for 3 h, to reaching exponential growth phase. Bacteria were collected by centrifugation (4000 g, 5 min) and resuspended in 1 mL of PBS (pH = 7.4). *E. coli* colonies were quantified by optical density (OD) at 540 nm (OD = 10^9^ bacteria/mL). 

A known quantity of bacteria (10^7^ CFU mL^−1^) were then added to empty nanoparticles or Essential oils-loaded nanoparticles in a total volume of 1 mL and incubated for 24 h at 37 °C under 150 rpm. After this incubation, serial dilutions of each sample, were prepared and distributed in agar plates. The number of CFUs was determined after 24 h of incubation at 37 °C.

We used the same protocol for studying the antimicrobial effect of our nanoparticles in fresh orange juice after 24 h and one week incubation with *E. coli.* Fresh orange juice inoculated with *E. coli* (10^7^ CFU mL^−1^) was treated with empty nanoparticles and with nanoparticles loaded-OEO in a total volume of 1 mL and incubated for 24 h at 37 °C under 150 rpm. Next, serial dilutions were spread on agar plates and the number of CFUs was determined after 24 h of incubation. Finally, the experiment was repeated after one week of samples storage at room temperature (25°C). 

## 3. Results and Discussions

As generally reported in numerous research articles, special attention has been dedicated to the encapsulation efficiency and to drug delivery, but the colloidal characterization of the prepared dispersions has been totally neglected. Then, the major aim of the work is to examine deeply the colloidal characterization of all prepared dispersions as below discussed.

### 3.1. Hydrodynamic Size and Zeta Potential as Function of pH and Salinity

**Figure 1:**
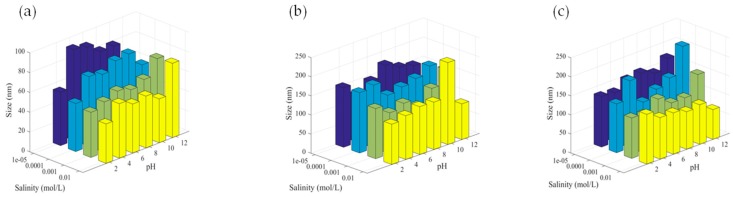
Hydrodynamic sizes of empty nanoparticles (Nps) (**a**), bergamot essential oil nanoparticles (BEONps) (**b**), sweet orange essential oil nanoparticles (OEONps) (**c**) as a function of pH and salinity.

The hydrodynamic diameter of all prepared dispersions was measured using light scattering in highly diluted conditions in the presence of NaCl salt. The zeta potential was deduced from the measured electrophoretic mobility in the given conditions (salinity and pH). The obtained hydrodynamic sizes of all samples are reported in Figure 1 for the three samples (EMPTY, BEO and OEO Nps) as a function of pH and salinity. For empty nanoparticles, the hydrodynamic size was found to be between 50 to 90 nm. Whereas, for nanoparticles containing essential oil, the hydrodynamic size was found to be higher ranging from 120 to 150 nm. This increase can be attributed to the swelling of polymer nanoparticles by the essential oil. It is worth mentioning that the essential oil is not miscible in water, and low solubility of the Eudragit polymer in these essential oils is possible. Then, large particle size can be attributed to the swelling of Eudragit polymer by the essential oil, leading not only to large particles but also large size distributions compared to empty nanoparticles.

Regarding the effect of pH and salinity, no noticeable tendency can be deduced from the investigated study. However, we can conclude that with or without essential oil, the obtained dispersions are of good colloidal stability since no aggregation phenomena have been observed in the investigated pH and salinity ranges. 

**Figure 2:**
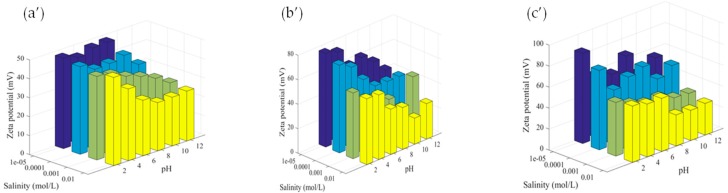
Zeta potential of empty Nps (**a’**), BEONps (**b’**) and OEONps (**c’**) as a function of pH and salinity.

Zeta potential of all dispersions measured as a function of pH and salinity is reported in Figure 2. As can be seen from these subfigures, the zeta potential is always in a positive range, irrespective of pH and salinity, reflecting the cationic character of the particle surface. This is attributed to the presence of the quaternary ammonium group on the particle surface, leading to the non-pH sensitive polymer. As expected, the increase in pH induces low decreases of zeta potential but remains positive in the investigated pH range. This slight decrease can be principally attributed to hydroxide compound concentration surrounding the particle surface inducing not only shift in the sleeping plan but also a slight screening of surface charges on the particles. 

The effect of salinity on zeta potential was found to slightly decrease irrespective of pH. This behavior can be attributed to the screening effect of surface charges by the added ions, leading consequently to low zeta potential as theoretically expected. A similar tendency has been reported not only for classical polymer particles, such as polystyrene, but also for more complex colloids [30,31]. It is worth noting that a slight aggregation phenomenon has been observed at high salinity and at pH = 12. This can be attributed to the low colloidal stability of the particles when the zeta potential is decreased. As a general tendency, the colloidal stability of surfactant-free dispersion decreases when the pH is close to the isoelectric point (IEP).

### 3.2. Colloidal Stability Study

In food science and drug delivery, the colloidal stability of any prepared or formulated dispersion is of paramount importance. In food science, the particles of the capsules should be well dispersed, and no marked sedimentation is highly appreciated. In drug delivery, stable colloidal dispersions are well appropriate for administration, blood circulation, and then bioavailability. 

**Figure 3:**
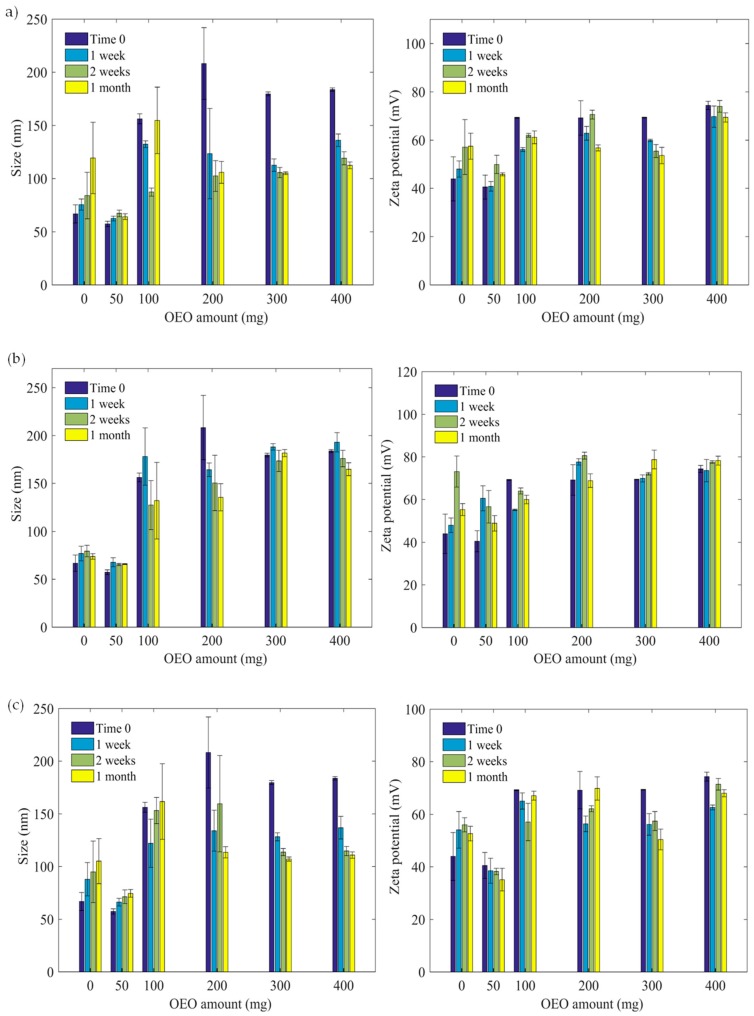
(**a**) Size and zeta potential at 25 °C for orange essential oil, (**b**) 4 °C, (**c**) 40 °C.

**Figure 4:**
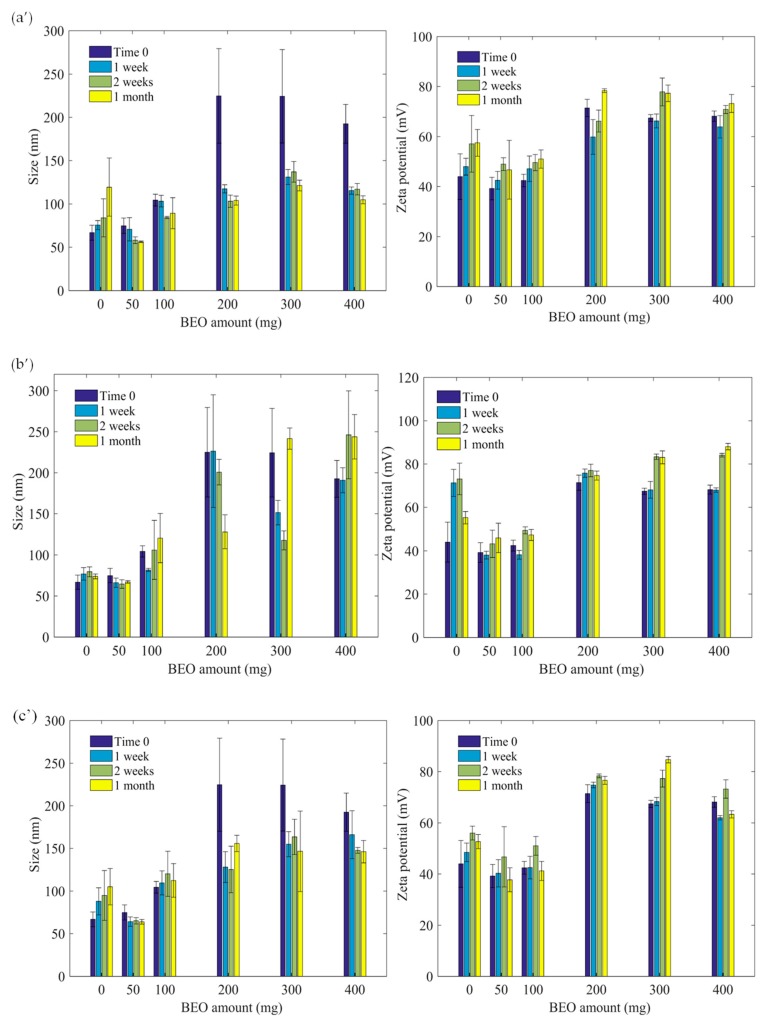
(**a’**) Size and zeta potential at 25 °C for bergamot essential oil, (**b’**) 4 °C, (**c’**) 40 °C.

In order to point out if the prepared dispersions are stable, hydrodynamic size and zeta potential as a function of temperature (4, 25, 40 °C) and time storage (one week, two weeks, one month) (Figure 3 and Figure 4) were studied.

As can been seen from the graphs (Figure 3), at 25 °C, for OEONPs, the average hydrodynamic size decreases as a function of time except for nanoparticles containing 50 mg of OEO at 4 °C which exhibit constant size irrespective of time in the investigated range. Whereas, the hydrodynamic particles size of empty particles increases at 37 °C and also for particles containing 50 and 100 mg of orange essential oil, but the size slightly decreases for particles loaded with 200, 300 and 400 mg of essential oil. On the basis of these data, we can state that the orange containing nanoparticles are stable when preferably stored at 4 °C, which is also suitable for food preservation. Regarding the zeta potential measured as a function of essential oil content, incubation time and temperature, the determined values are almost constant and above +38 mV revealing medium surface charge density, but sufficient to maintain appreciable colloidal stability in the investigated conditions.

As for orange essential oil containing nanoparticles, bergamot loaded nanoparticles dispersions have been also analyzed in the same conditions, and the obtained results are reported in Figure 4.

**Figure 5:**
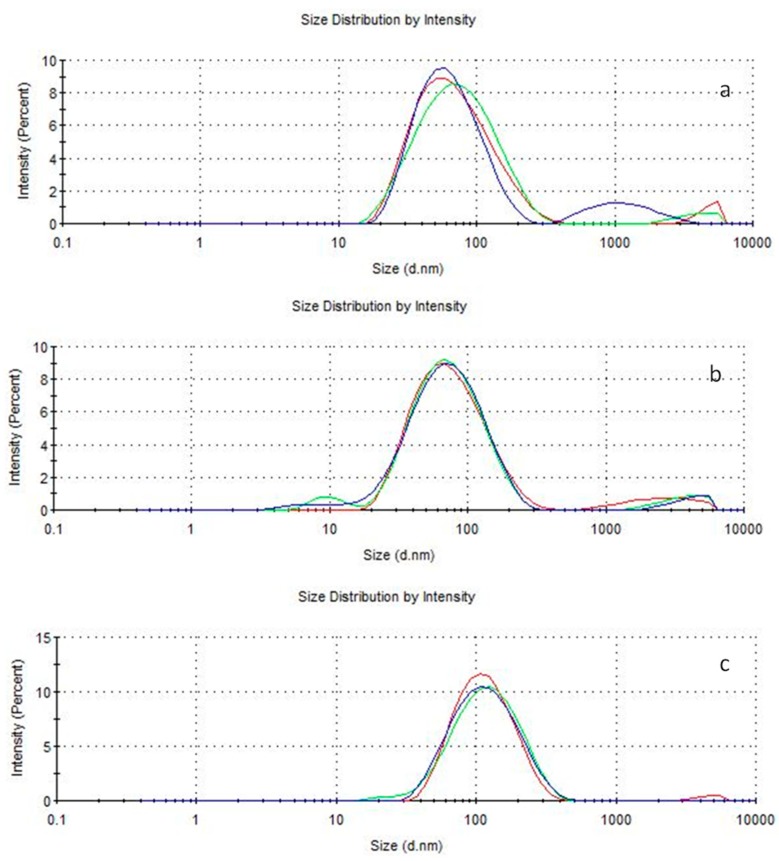
Particle size distribution of empty (**a**), BEO-loaded Nps (**b**), OEO-loaded Nps (**c**) after one week storage at 4 °C.

At 25 °C, the hydrodynamic sizes increase principally for empty nanoparticles, are moderately constant for nanoparticles loaded with 50 and 100 mg of bergamot essential oil, and dramatically decrease after one week for the nanoparticles containing 300 and 400 mg of essential oil. At 4 °C incubation condition, the hydrodynamic sizes are constant irrespective of time for empty and for nanoparticles loaded with 50 mg of BEO. Whereas, for other bergamot containing particles, the hydrodynamic size is stable after one week. At 40 °C, the sizes increase for empty nanoparticles and are almost constant for nanoparticles loaded with 50 and 100 mg of essential oil. For nanoparticles loaded with 200, 300, and 400 mg of essential oil, the hydrodynamic size was found to decrease. Zeta potential values are about constant over time at all temperatures except for 300, and 400 mg loaded nanoparticles at 4 °C. It is worth noticing that the pH of the dispersions is stable and found to be between five and six. Particle size distribution was investigated during storage time in studied condition. As can be seen in Figure 5, in which only an example for empty nanoparticles (a), BEO-loaded Nps (b) and OEO-loaded Nps (c) is reported, a narrow size distribution has been observed.

### 3.3. Fluorescence Microscopy Analysis

**Figure 6:**
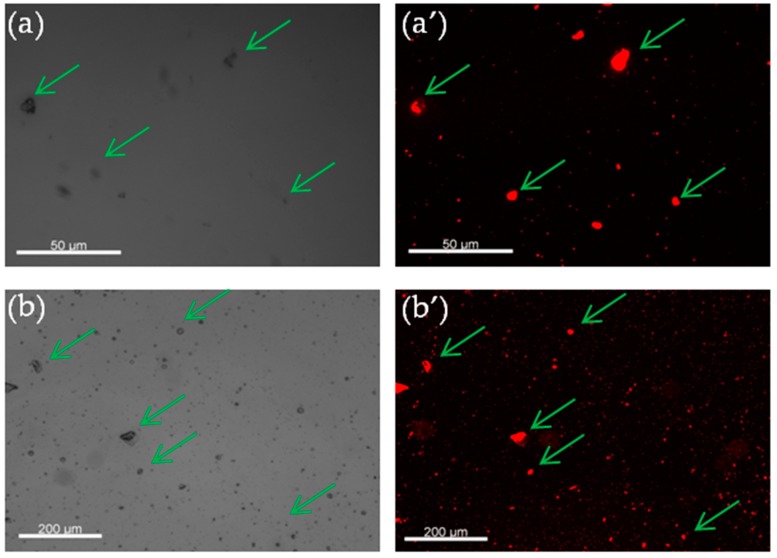
Fluorescence microscope images of fluorescent loaded nanoparticles. (**a**) Orange essential oil loaded nanoparticles with green light. (**a’**) Orange essential oil loaded nanoparticles with white light. (**b**) Bergamot essential oil loaded nanoparticles with green light. (**b’**) Bergamot essential oil loaded nanoparticles with white light.

In order to observe qualitatively if the essential oil is homogeneously encapsulated in polymer particles, a fluorescent molecule and fluorescence microscopy were used. First, a non-water-soluble fluorescent molecule was dissolved in essential oil and then encapsulated using the same process. For this reason, a fluorescent agent was added into the BEO/OEO before their encapsulation. This allows visualization of encapsulated EOs in nanoparticles during observation by fluorescence microscopy (see Figure 6). As it can be evidenced from the analysis of all the images (a and a’, b and b’), all the observed nano-objects in white light are also fluorescent under fluorescence microscopy, demonstrating that all the prepared nanoparticles contain essential oil.

### 3.4. Scanning Electron Microscopy Analysis 

**Figure 7:**
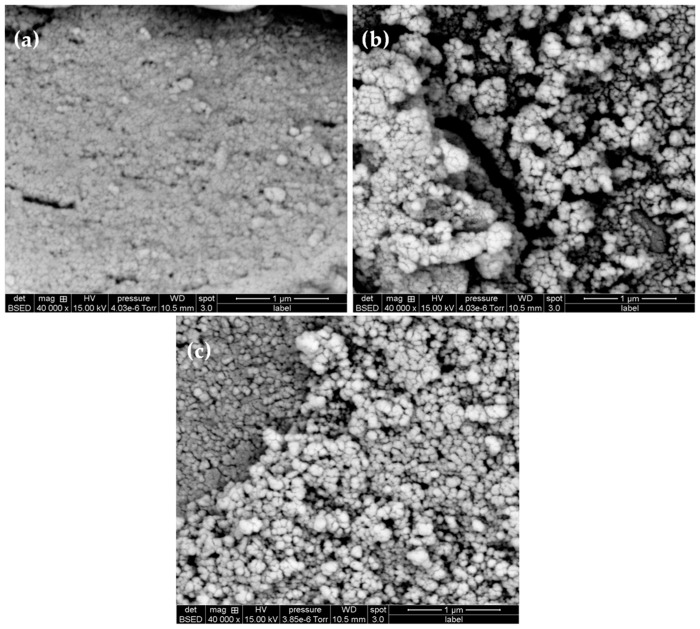
SEM images of (**a**) empty particles and nanocapsules containing (**b**) orange essential oil and (**c**) bergamot essential oil.

The prepared dispersions have been imaged by scanning electron microscopy (SEM) in order to examine the size and size homogeneity and also the particle morphology. As illustrated in Figure 7, it is difficult to obtain good images for two main reasons: (i) The low density of the used polymer which is poly(methyl methacrylate) based and (ii) the low particle size. In fact, it is well-known that the viewing of nanosized polymer particles is not easy compared to inorganic nanoparticles. We basically deduced from SEM images that the prepared dispersions are submicron in size and even below 100 nm. Regarding the morphology, spherical-like particles have been identified but it was impossible to determine the exact shape. 

### 3.5. Atomic Force Microscopy (AFM)

**Figure 8:**
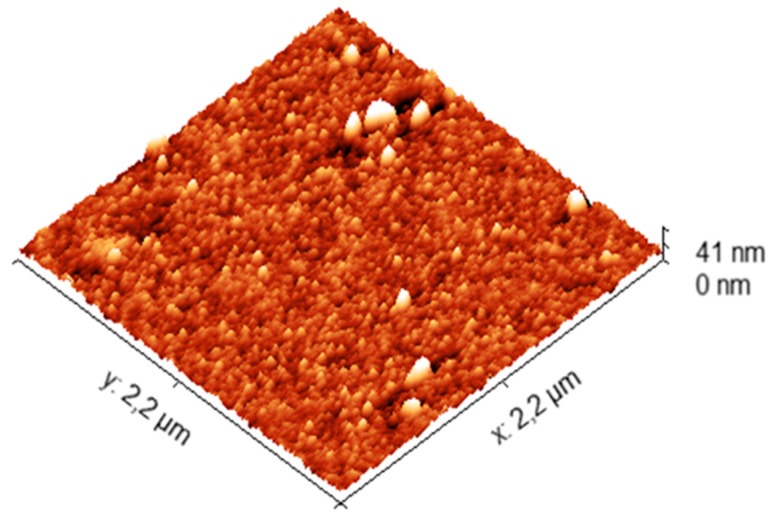
Atomic Force Microscopy imaging of empty polymer particles.

In order to confirm the particle size and also the particle morphology, atomic force microscopy was used and the obtained images are reported in Figure 8 shows the topography of the deposited empty polymer particles on silicon nitride support which is extremely smooth and presents a very low roughness [32]. As clearly evidenced from AFM images, very well distinguishable and homogeneous nanoparticles distribution with an average roughness of 51.68 pm revealing that the particle size is in a nanosize range. This is in good agreement with the value deduced from light scattering analysis. Essential oils containing polymer nanoparticles are not deeply analyzed by AFM since it was pointed out that it is hard to deduce exactly the particle size and size distribution of such polymer nanoparticles, but only tendency can be reported and discussed. 

### 3.6. Gas Chromatography Analysis of Sweet Orange and Bergamot Essential Oils

Chemical analysis of essential oils is not really easy and special equipment and references are necessary in order to point out the exact chemical composition of any oil and essential oil. Gas chromatography analysis of the oils for five components indicated that limonene was the more abundant component in the tested oils (Table 1).

### 3.7. Encapsulation Efficiency

The encapsulation efficiency of orange and bergamot essential oils has been investigated as a function of the essential oil initial amount. As expected, the encapsulation amount of essential oil increases with the increasing of the used initial amount before reaching plateau like the value of 25 mg/mg of polymer. Such high encapsulation efficiency is possible because the used essential oils are less miscible with water but miscible with acetone used as a polymer solvent. It is worth mentioning that essential oil is not a good solvent for the polymer, consequently it is not possible to discuss any interpretation based on the reach of 100% of encapsulation efficiency due to the possible solubility of essential oil in the acetone-water mixture. In short, encapsulation efficiency of bergamot essential oil was found to be between 28% and 84%, while for orange essential oil it was ranged from 48% to 96% (see Table 2).

### 3.8. Antimicrobial Activity

**Figure 9:**
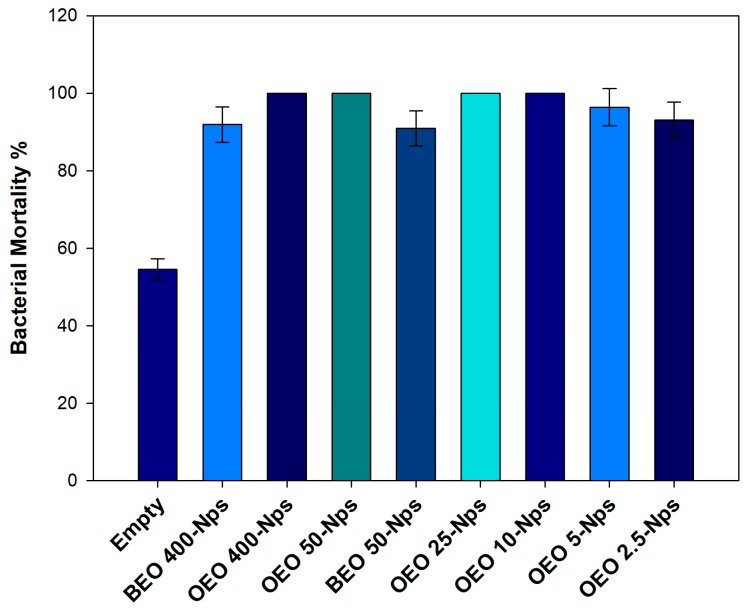
Bacterial mortality of *E. coli* after 24 h incubation at 37 °C with empty NPs and NPs-loaded EOs. Data represented as a percentage are the results of six different tests compared to the control (untreated *E. coli*).

The antimicrobial activity of empty nanoparticles and essential oil-loaded nanoparticles was investigated using *E. coli* mortality. Empty nanoparticles were found to have low antimicrobial activity, and the observed activity is related to the cationic property of the particle surface induced by Eudragit RS-100 polymer. Whereas, the antimicrobial activity of encapsulated essential oil in nanocapsules was found to be around 90% irrespective of essential oil nature and essential oil amount. This can be attributed to the combined effect of the cationic character of the particles and the presence of essential oils [33]. It is interesting to notice that the lowest amount of essential oil used leads to a good antimicrobial activity, as reported in Figure 9. As a general tendency, orange essential oil seems to have higher antimicrobial activity compared to bergamot essential oil. 

**Figure 10:**
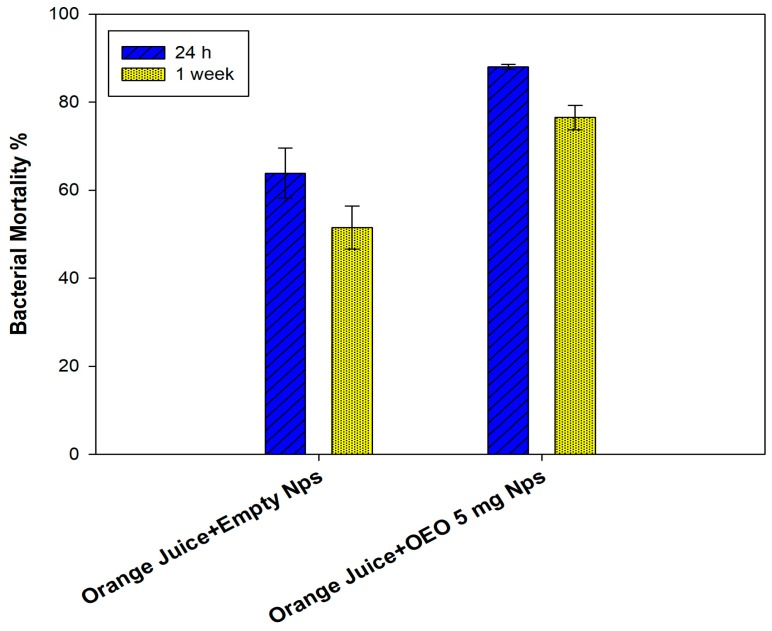
Bacterial mortality of *E. coli* after 24 h and after one week incubation at 25 °C with empty NPs and NPs-loaded 5 mg of OEO. Data represented as a percentage are the results of six different tests compared to the control (untreated *E. coli*).

Due to the high effectiveness of orange essential oil, the real effect on non-sterilized orange juice preservation was investigated as a function of time and for the lowest essential oil amount. Empty particles have almost a constant effect irrespective of incubation with a slight decrease after one week. Whereas, in the case of nanocapsules containing essential oil, the antibacterial activity is more pronounced with 90% of bacterial mortality as expected and a slight decrease (80%) after one week of incubation (see Figure 10).

## 4. Conclusions

Bergamot and orange essential oils-loaded polymer nanoparticles were prepared via nanoprecipitation method using a cationic poly(methyl methacrylate)-based polymer. The colloidal properties of all prepared nanoparticles were investigated in terms of size, size distribution, and colloidal stability. The resulting tendencies show submicron positively charged particles with good colloidal stability and narrow size distributions. The antimicrobial activity of all prepared dispersions against *E. coli* was investigated as a function of essential oil amount and incubation time. Empty particles show medium activity due to the cationic character of the used polymer, whereas the effect of nanocapsules containing essential oil is more pronounced and found to be high for orange essential oil. The real effect of the encapsulated orange essential oil has been investigated using fresh orange juice and found to enhance the period of its conservation. This promising result combining the use of cationic nanocapsules and orange essential oil can be extended to a good edible polymer for real applications in food preservation.

## Figures and Tables

**Table 1:** Analysis of oils by gas chromatography (only the main compounds responsible for antibacterial were reported).

Component	Bergamot (%)	Sweet Orange (%)
Limonene	20.19	95.53
Myrcene	-	2.53
Linalool	8.08	-
Linalyl acetate	5.92	-
γ-terpinen	5.15	-

**Table 2:** Encapsulation efficiency and essential oil entrapment/mg polymer for all nanoparticles (data ± standard deviation). SD = standard deviation.

Sample	Essential Oil Amount (mg/mL)	Entrapment Efficiency % (± SD)	Essential Oil Entrapped in mg/mg of Polymer
5 mg OEO	0.1	56 ± 0.2	0.014
10 mg OEO	0.2	62 ± 0.2	0.031
25 mg OEO	0.5	60 ± 1.2	0.075
50 mg OEO	1	48 ± 0.8	0.12
100 mg OEO	2	80 ± 1.5	0.4
200 mg OEO	4	74 ± 2.3	0.74
300 mg OEO	6	96 ± 1.8	1.44
400 mg OEO	8	63 ± 0.9	1.26
50 mg BEO	1	28 ± 0.7	0.07
100 mg BEO	2	46 ± 0.5	0.23
200 mg BEO	4	84 ± 0.8	0.84
300 mg BEO	6	68 ± 2.8	1.02
400 mg BEO	8	58 ± 1.5	1.16
